# Characterization and photocatalytic study of tantalum oxide nanoparticles prepared by the hydrolysis of tantalum oxo-ethoxide Ta_8_(μ_3_-O)_2_(μ-O)_8_(μ-OEt)_6_(OEt)_14_

**DOI:** 10.3762/bjnano.5.121

**Published:** 2014-07-18

**Authors:** Subia Ambreen, N D Pandey, Peter Mayer, Ashutosh Pandey

**Affiliations:** 1 Department of Chemistry, Motilal Nehru National Institute of Technology, Allahabad, 211004, India; 2 Department Chemie und Biochemie, Universität München, Butenandtstraße 5–13, 81377 München, Germany

**Keywords:** bandgap, tantalum-oxo-ethoxide, Tauc plot, tantalum pentoxide (Ta_2_O_5_)

## Abstract

Ta_8_(μ_3_-O)_2_(μ-O)_8_(μ-OEt)_6_(OEt)_14_ (**1**) was obtained by the controlled hydrolysis of tantalum ethoxide Ta(OEt)_5_ in the presence of ammonia. Compound **1** is considered as the intermediate building block in the sol–gel polymerization of Ta(OEt)_5_. Further hydrolysis of compound **1** yielded nanoparticles of Ta_2_O_5_, which were characterized by various techniques such as TGA-DTA-DSC, UV–vis DRS, XRD, SEM, TEM, particle size analyzer (DLS) and the Brunauer–Emmett–Teller (BET) method. The band gap of the particles was calculated by using the Tauc plot. The photocatalytic activity of Ta_2_O_5_ nanoparticles was tested by the degradation of the organic dye rhodamine B.

## Introduction

Metal alkoxides, being excellent precursors in the sol–gel process for preparation of metal oxides have attained huge attention of researchers. Several attempts have been made in order to modify the highly moisture sensitive metal alkoxides into less sensitive species [1–4] as precursors for metal oxides with new and better properties. However, due to the fast kinetics of the hydrolysis and condensation reactions in the sol–gel route, relatively little information is available concerning the progressive structural evolution in the transition metal oxide system in general. But sometimes new species, metal oxo-alkoxides [5–9], are obtained which have been known to be the direct molecular precursors for oxide phases in sol–gel technology. These oxo-species being treated as intermediates between the metal alkoxides and the metal oxides are very significant as they clearly indicate the route for the formation of oxides through hydrolysis. It is suggested that the hydrolysis of a metal alkoxide starts with the formation of a hydroxo derivative, which then forms the oxo derivative in a condensation step (Equation 1 and Equation 2).

[1]
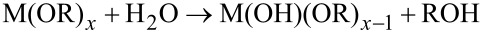


[2]
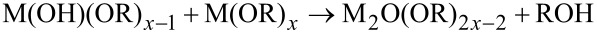


The investigation of the hydrolysis of titanium alkoxides showed that the reactions were very fast. Therefore the initial hydroxo compounds were not isolated [10]. Similar results were obtained for alkoxides of zirconium [11], tin(IV) [12], and uranium(V) [12]. Condensation can also occur even before hydrolysis via ether elimination between alkoxy groups leading to the formation of oxo bridges. The smaller size of μ-oxo ligands coupled with the tendency of metal centers for coordination expansion, favors the condensation via ether elimination.

Oxo-alkoxides, being less reactive toward hydrolysis and condensation, are more stable than the corresponding alkoxides. They are generally observed for large and electropositive metals. Oxo-alkoxides are normally made of edge sharing MO_6_ octahedra. Usually the physical properties of metal oxo-alkoxides are decided by the degree of hydrolysis and the nature of the alkyl group. There is a tendency for lower volatility and solubility with higher degrees of hydrolysis and, therefore, oligomerization. Amongst the transition metal oxides, Ta_2_O_5_ has attracted growing interest due to its distinct properties such as large ion diffusion coefficient and high electrochromic reversibility, high dielectric constant, high refractive index, high chemical stability, large band gap [13–15] and photocatalytic activity for overall water decomposition and organic pollutant degradation [16–21].

The present work deals with the study of the controlled hydrolysis of tantalum ethoxide in the presence of ammonia and to prepare tantalum pentaoxide nanoparticles. In this process the stable intermediate tantalum oxo-ethoxide with composition Ta_8_(μ_3_-O)_2_(μ-O)_8_(μ-OEt)_6_(OEt)_14_ (**1**) was isolated. When **1** was subjected to further hydrolysis it yielded nanoparticles of tantalum oxide after calcination at 750 °C for four hours. The photocatalytic activity of Ta_2_O_5_ nanoparticles was studied over the degradation of organic dye rhodamine B (RhB).

## Results and Discussion

Tantalum penta-ethoxide was dissolved in toluene and with the aim to examine the effect of hydrolysis in basic medium, wet ammonia gas was purged into it with continuous stirring. After 1 h at pH 8.0, a white solid was formed which was separated, re-dissolved in toluene and kept at low temperature for crystallization to give compound **1** as white shiny crystals in two days. The formation of tantalum oxo-alkoxide can be described as a result of following reactions (Equation 3 and Equation 4).

[3]
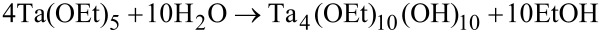


[4]
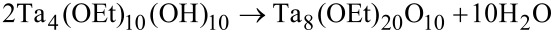


^1^H NMR of compound **1** shows many sets for ethoxy groups suggesting the presence of different types of ethoxy groups such as cis pairs of terminal OEt ligands, single terminal OEt ligands and μ-OEt groups.

### Crystal structure

**Figure 1:**
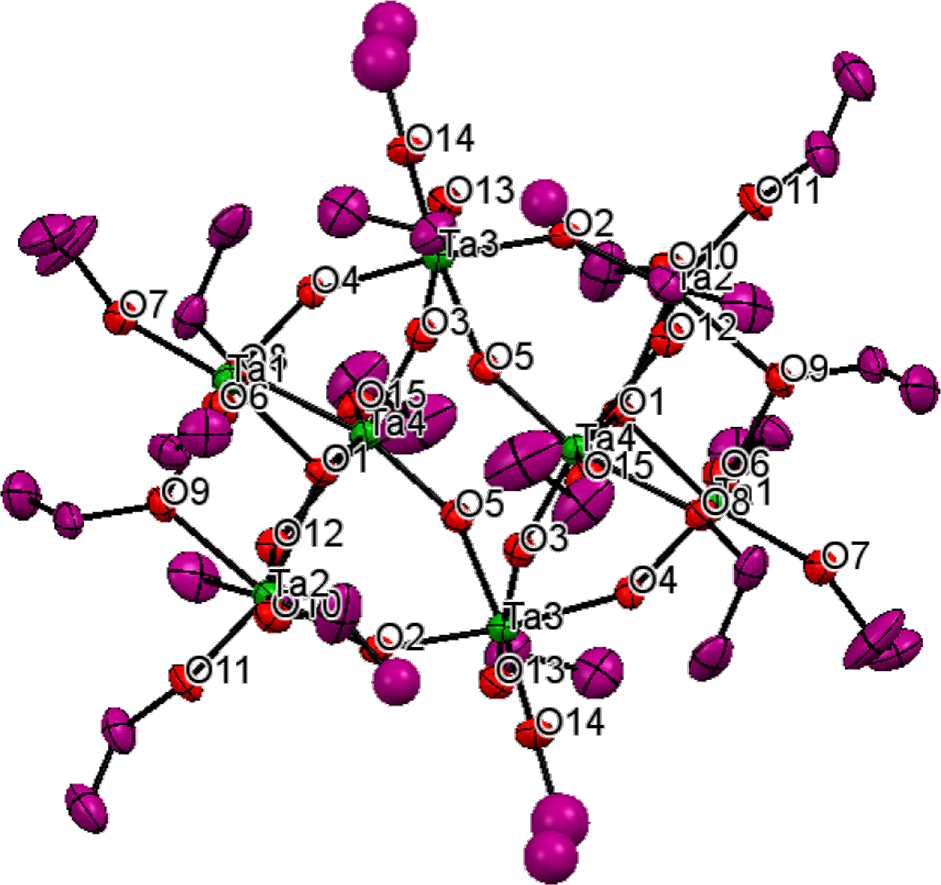
ORTEP representation of the molecular structure of **1** in the crystal (hydrogen atoms are omitted for clarity).

Compound **1** was found in the monoclinic space group *P*21/*n* with *Z* = 2. The molecular structure (Figure 1) consists of a centrosymmetric unit of formula Ta_8_(μ_3_-O)_2_(μ-O)_8_(μ-OEt)_6_(OEt)_14_ (**1**). Each molecule has two Ta_3_(μ_3_-O)-(μ-OEt)_3_(OEt)_5_ units linked with two Ta(μ-O)_4_(OEt)_2_ moieties by four μ-oxo ligands. All of the tantalum atoms display distorted octahedral configurations. Six tantalum atoms are bonded to cis pairs of terminal ethoxy ligands and the other two are bonded to single ethoxy ligands. The terminal ligands are in trans position to the μ_3_- and μ-oxo atoms. The ethoxy groups are of two types. Out of the twenty ethoxy groups, fourteen are terminal while six (O8, O9, O12 and O8*, O9*, O12*) bridge the tantalum atoms. The tantalum atoms have two environments. Four tantalum atoms are coordinated to two terminal ethoxy, two bridging ethoxy, one μ-O and one μ_3_-O ligands while two tantalum atoms are coordinated with two terminal ethoxy and four μ-O groups. The TaO_6_ octahedra are distorted, resulting in O–Ta–O angles, which differ from those for a regular octahedron. Some bond lengths and bond angles are presented in Table 1 and Table 2 respectively. Ta–O bond lengths for the terminal ethoxy ligands are shorter (av. 1.897 Å) than the bridging ethoxy ligands (av. 2.153 Å). The μ-oxo bridges are shorter than the μ_3_-O bond lengths (approx. 2.0585 Å). The μ_3_-oxo atom, O1 is ligated in pyramidal manner to three tantalum atoms with an average bond angle of 108.87°. The bond dimensional data are in accordance with previously reported values [5,9]. Full crystallographic details have been deposited to the Cambridge Crystallographic Data Centre. Copies of the data can be obtained free of charge on request from the CCDC, 12 Union Road, Cambridge CB2 1EZ, UK; fax: (+44) 1223-336-033; or e-mail: deposit@ccdc.cam.ac.uk or http://www.ccdc.cam.ac.uk/conts/retrieving.html quoting the deposition number CCDC 951412 for **1**.

**Table 1:** Selected bond lengths.

bond	length	bond	length

O1–Ta1	2.0629(1)	O7–Ta1	1.8711
O1–Ta2	2.0345(1)	O8–Ta1	2.1061(1)
O1–Ta4	2.0719(1)	O8–Ta4	2.1556(1)
O2–Ta2	1.8577	O9–Ta1	2.1533(1)
O2–Ta3	1.9928	O9–Ta2	2.1319(1)
O3–Ta3	2.0039(1)	O10–Ta2	1.8928
O3–Ta4	1.8544(1)	O11–Ta2	1.8669
O4–Ta3	2.0167(1)	O12–Ta2	2.1149(1)
O4–Ta1	1.8411	O12–Ta4	2.1725(1)
O5–Ta3	2.0238(1)	O13–Ta3	1.9083
O5–Ta4	1.8455	O14–Ta3	1.8783(1)
O6–Ta1	1.8927(1)	O15–Ta4	1.8671

**Table 2:** Selected bond angles.

bond	angle	bond	angle

Ta1–O1–Ta2	110.85	O9–Ta2–O11	89.83
Ta1–O1–Ta4	107.48	O9–Ta2–O12	87.09
Ta2–O1–Ta4	108.28	O10–Ta2–O11	100.04
Ta2–O2–Ta3	144.86	O10–Ta2–O12	167.96
Ta3–O3–Ta4	145.34	O11–Ta2–O12	91.4
Ta3–O4–Ta1	144.33	O2–Ta3–O3	90.31
Ta3–O5–Ta4	144.09	O2–Ta3–O4	173.29
Ta1–O8–Ta4	102.94	O2–Ta3–O5	85.31
Ta1–O9–Ta2	103.87	O2–Ta3–O13	96.48
Ta2–O12–Ta4	101.83	O2–Ta3–O14	89.15
O1–Ta1–O6	99.79	O3–Ta3–O4	83.84
O1–Ta1–O7	154.81	O3–Ta3–O5	84.94
O1–Ta1–O8	72.52	O3–Ta3–O13	168.36
O1–Ta1–O9	71.58	O3–Ta3–O14	95.05
O1–Ta1–O4	92.52	O4–Ta3–O5	90.89
O6–Ta1–O7	97.78	O4–Ta3–O13	88.77
O6–Ta1–O8	170.71	O4–Ta3–O14	94.62
O6–Ta1–O9	87.55	O5–Ta3–O13	86.2
O6–Ta1–O4	95.44	O5–Ta3–O14	174.46
O7–Ta1–O8	88.03	O13–Ta3–O14	94.49
O7–Ta1–O9	91.35	O3–Ta4–O15	101.71
O7–Ta1–O4	103.66	O3–Ta4–O1	91.83
O8–Ta1–O9	85.07	O3–Ta4–O5	101.59
O8–Ta1–O4	90.18	O3–Ta4–O8	88.23
O9–Ta1–O4	164.11	O3–Ta4–O12	161.79
O1–Ta2–O2	93.37	O15–Ta4–O1	157.42
O1–Ta2–O9	72.57	O15–Ta4–O5	101.66
O1–Ta2–O10	94.28	O15–Ta4–O8	90.91
O1–Ta2–O11	157.16	O15–Ta4–O12	91.7
O1–Ta2–O12	73.68	O1–Ta4–O5	93.07
O2–Ta2–O9	165.72	O1–Ta4–O8	71.34
O2–Ta2–O10	94.45	O1–Ta4–O12	71.76
O2–Ta2–O11	103.07	O5–Ta4–O8	161.98
O2–Ta2–O12	86.52	O5–Ta4–O12	87.48
O9–Ta2–O10	89.2	O8–Ta4–O12	79.2

Upon further subjecting to hydrolysis (pH 9.0), compound **1** initially gave a homogeneous gel which was heated to 80 °C before annealing at 750 °C to obtain Ta_2_O_5_ nanoparticles. The morphology and composition of Ta_2_O_5_ nanoparticles are greatly influenced by the pH value [22]. The agglomeration of nanoparticles is enhanced at low pH due to the fast rate of hydrolysis and acidic surface of Ta_2_O_5_ while at the pH values above 10, instead of Ta_2_O_5_, tantalate salts are formed [22].

### Particle size and distribution of Ta_2_O_5_ nanoparticles

**Figure 2:**
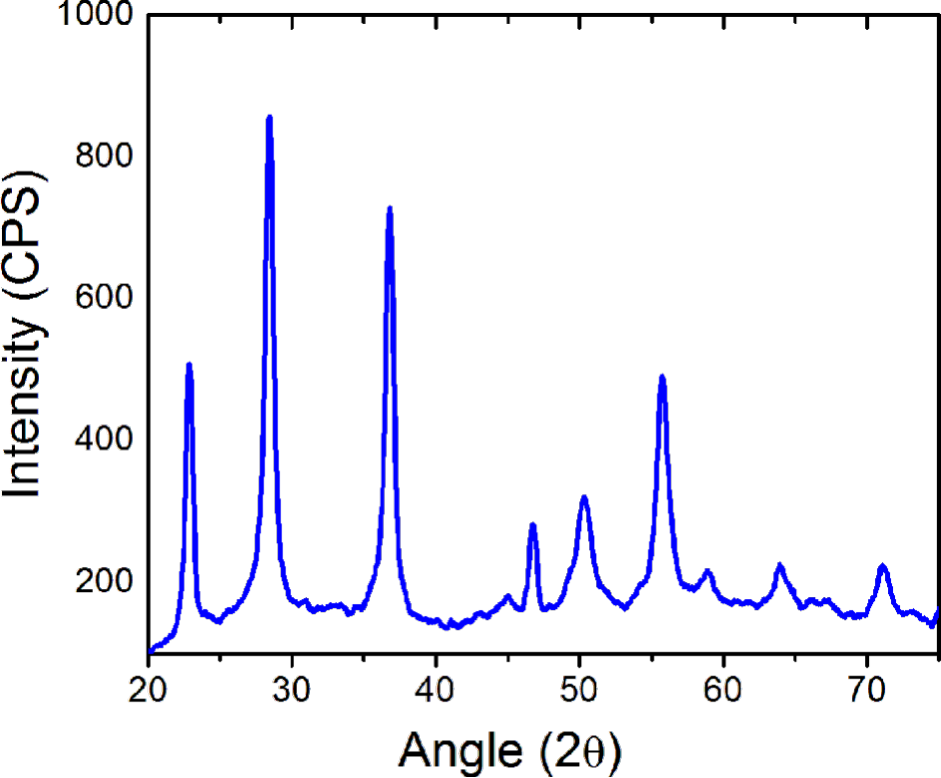
XRD pattern of Ta_2_O_5_ nanoparticles calcined at 750 °C for 4 h.

In order to study the size and distribution of the nanoparticles XRD, SEM, TEM and DLS measurements were carried out. Figure 2 shows the XRD patterns of calcined Ta_2_O_5_ nanoparticles. The peaks at 2θ values of 23.06, 28.62, 37.02, 46.94, 50.54, 55.72, 58.84, 64.18, and 71.30° revealed the Ta_2_O_5_ phase with an orthorhombic structure. The average crystallite size calculated from Scherrer formula was found to be 28 nm.

**Figure 3:**
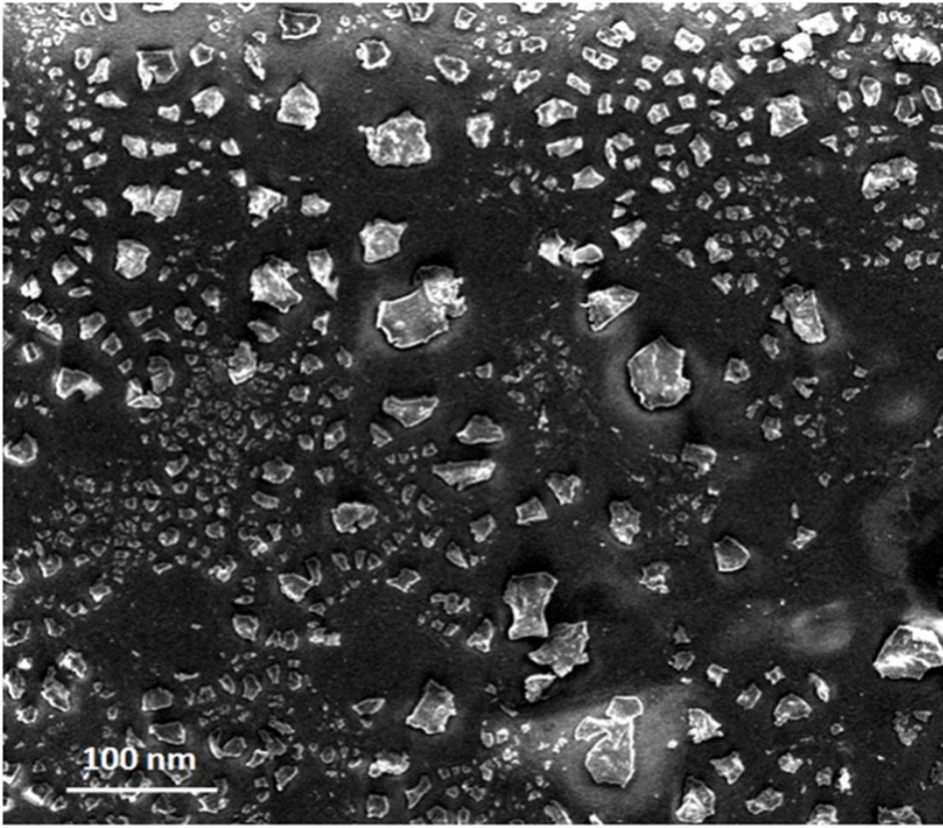
SEM image of Ta_2_O_5_ nanoparticles calcined at 750 °C for 4 h.

**Figure 4:**
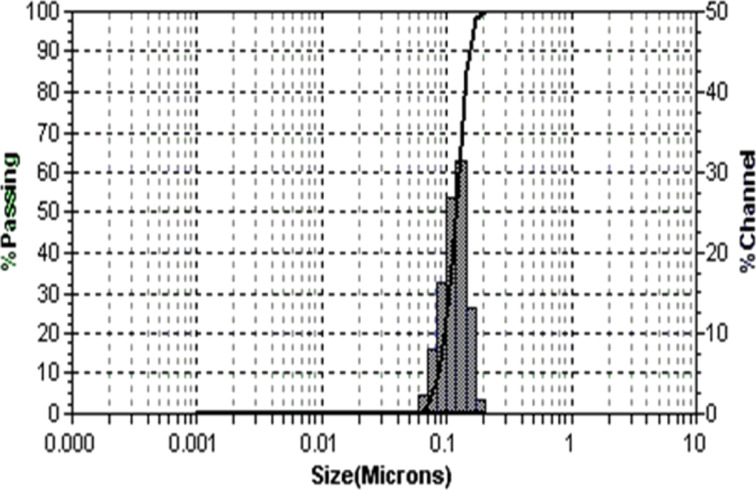
Size and distribution of TOPO-coated Ta_2_O_5_ nanoparticles in chloroform dispersion.

**Figure 5:**
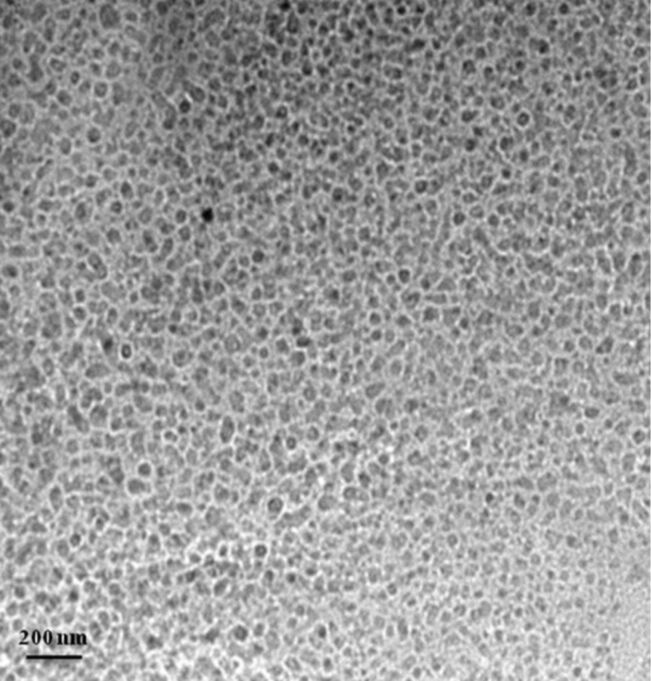
TEM image of the TOPO-coated Ta_2_O_5_ nanoparticles. The scale bar corresponds to 200 nm.

To reveal the morphology SEM image of the calcined Ta_2_O_5_ nanoparticles is shown in Figure 3. Evidently, Ta_2_O_5_ nanoparticles are irregularly shaped, agglomerated and exhibit a moderately uniform size distribution. The size distribution of the Ta_2_O_5_ nanoparticles was studied by DLS (Figure 4) in chloroform dispersion. Trioctylphosphine oxide (TOPO) was used as the surfactant for dispersing the nanoparticles in chloroform. It was found that the nanoparticles are almost uniformly distributed. TOPO is reported to fragment bigger nanoparticles or agglomerates into smaller ones. TOPO molecules attach to the metal oxide particle surface in such a manner that their hydrophobic surfaces point toward the solvent and render colloidal stability and uniformity of the particles in organic solvents [23]. The TOPO coated particles suspended in chloroform were precipitated by adding excess methanol followed by centrifuging at 2000 rpm and re-dispersed in chloroform. A TEM image of the so obtained particles is shown in Figure 5.

### Thermal analysis of the prepared Ta_2_O_5_ nanoparticles

**Figure 6:**
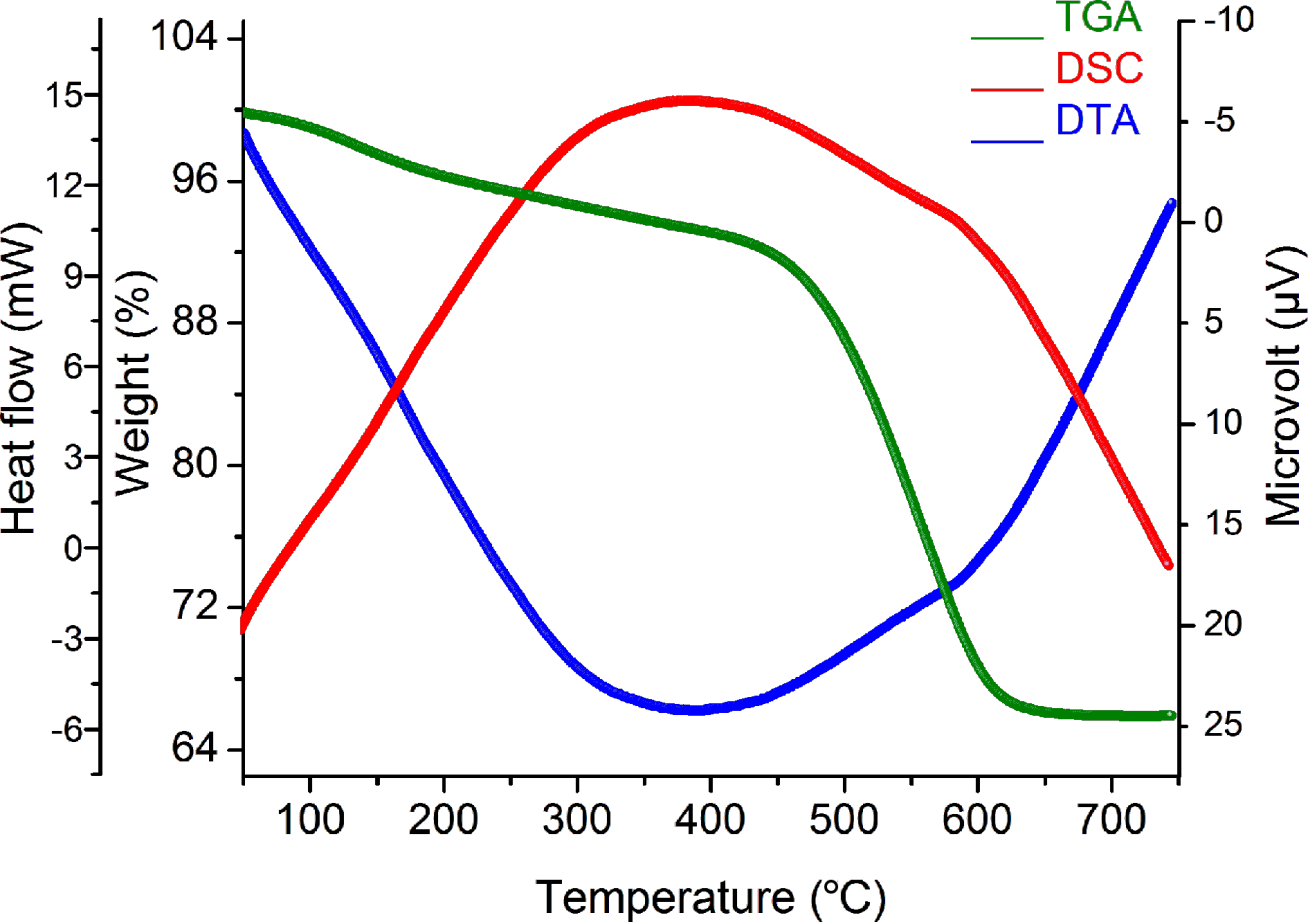
Thermogravimetry (TGA), differential thermal analysis (DTA) and differential scanning calorimetry (DSC) of the as-synthesized Ta_2_O_5_ nanoparticles.

Thermogravimetry, differential thermal analysis and differential scanning calorimetry (TG/DTA/DSC) with a heating rate of 10 °C/min in a static air atmosphere were used to study the thermal stability of the as-prepared (dried) photocatalyst with α-Al_2_O_3_ as the reference. Figure 6 shows the TG/DTA/DSC curves obtained from the dried gel of Ta_2_O_5_. The TGA graph shows a weight loss up to a temperature of 200 °C that is essentially attributed to dehydration. The decomposition of organic substances at 200–400 °C is caused by the decomposition of organic species inside the mesopores of the sample. Further the weight loss in the temperature range of 400–650 °C is due to a phase transition. An exothermic peak centered at 386.7 °C in the DTA curve supports the statements above. The observation is also supported by the DSC graph.

### Band-gap determination of the Ta_2_O_5_ nanoparticles

**Figure 7:**
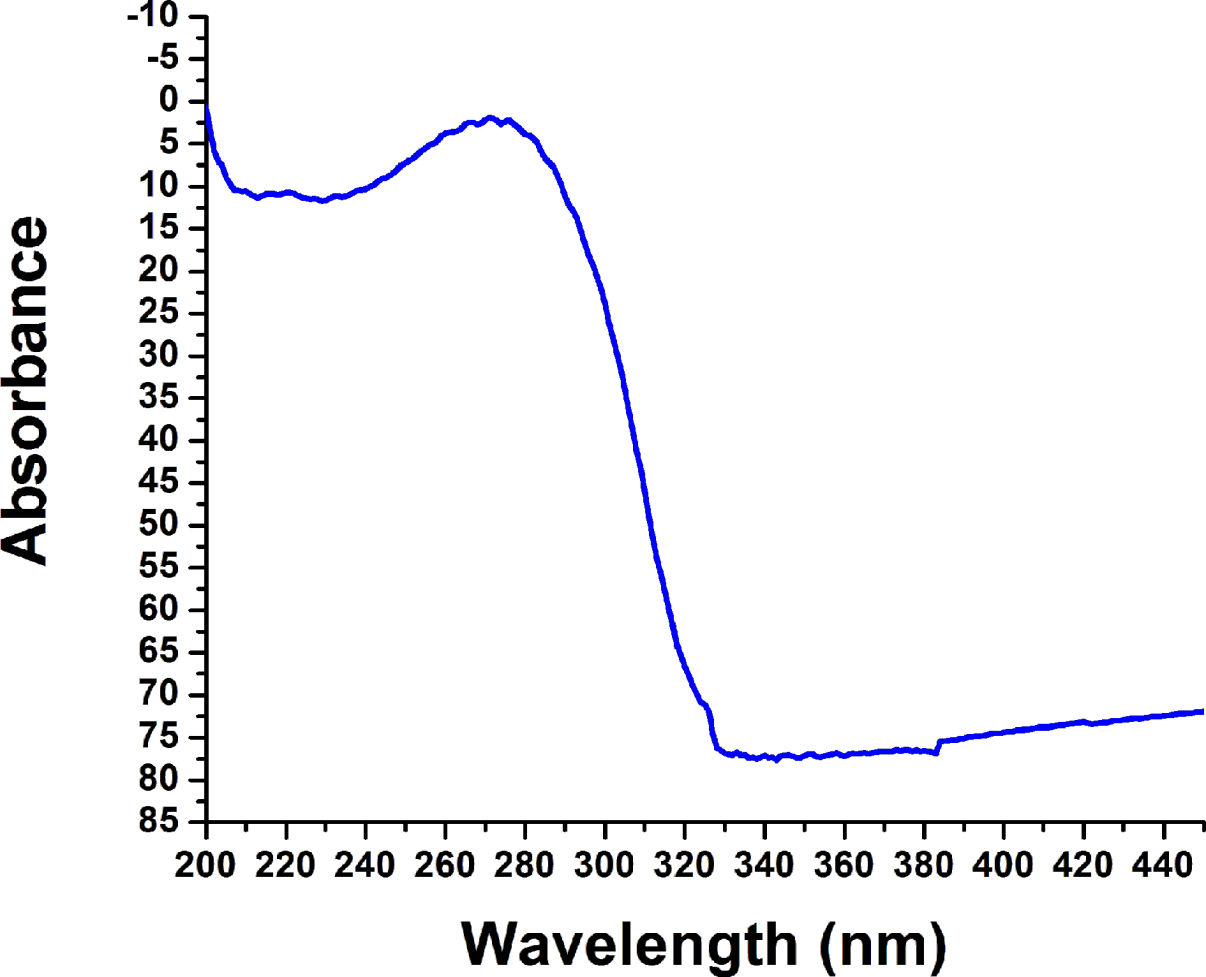
Solid state diffuse reflectance UV–vis spectra of Ta_2_O_5_ nanoparticles.

The band gap energy (*E*_g_) is a key feature of semiconductors that determines their applications in optoelectronics. The nanoparticles were pressed into thick pellets and subject to diffuse reflectance measurement, which was done with a UV–vis spectrophotometer, attached with integrating sphere to spatially integrate the radiant flux. The pellets were placed at the entrance port of the integrating sphere. The absorption spectrum of Ta_2_O_5_ nanoparticles is shown in Figure 7. The reflectance data was converted to the absorption coefficient *F*(*R*′) values according to the Kubelka–Munk remission function [24–26] (Equation 5),

[5]
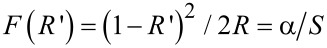


where α is the absorption coefficient (cm^−1^) and *S* is the dispersion factor. The absorption coefficient α is related to the incident photon energy by Equation 6:

[6]
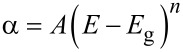


**Figure 8:**
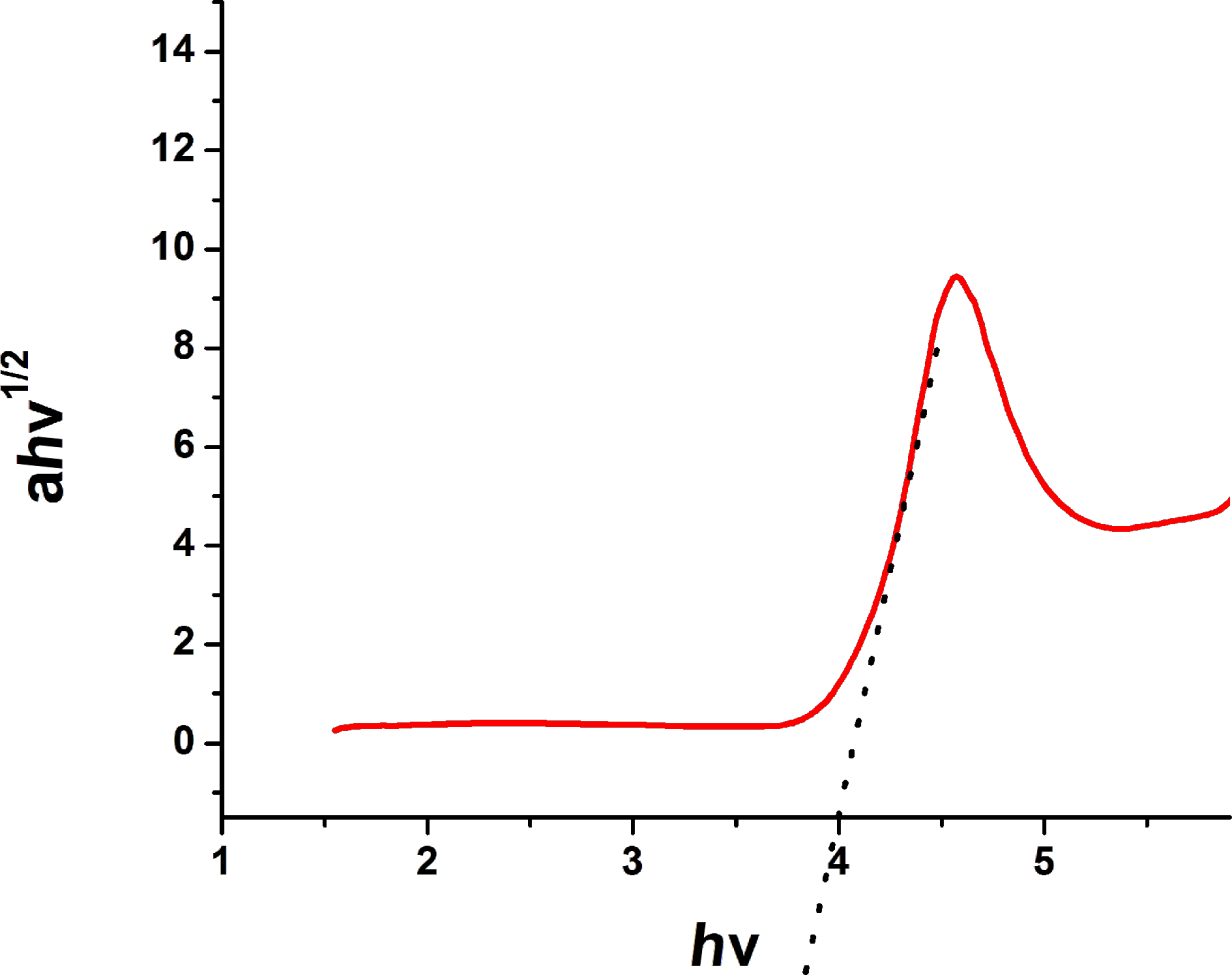
Calculation of band gap of Ta_2_O_5_ nanoparticles by Tauc plot.

*A* is a constant for the given material, *E* is the photon energy, *E*_g_ is the band gap energy and *n* is a constant of different values, 1/2, 3/2, 2 and 3, depending on the type of electronic transition, i.e., permitted/prohibited-direct or indirect transition. The band gap is calculated from a Tauc plot [27–30]. The band gap of the Ta_2_O_5_ nanoparticles as calculated from the extrapolation of the linear portion of the plot in the α*h*ν^1/2^ vs *h*ν graph to the abscissa (Figure 8) was found to be 3.9 eV.

### Brunauer–Emmett–Teller (BET) analysis

**Figure 9:**
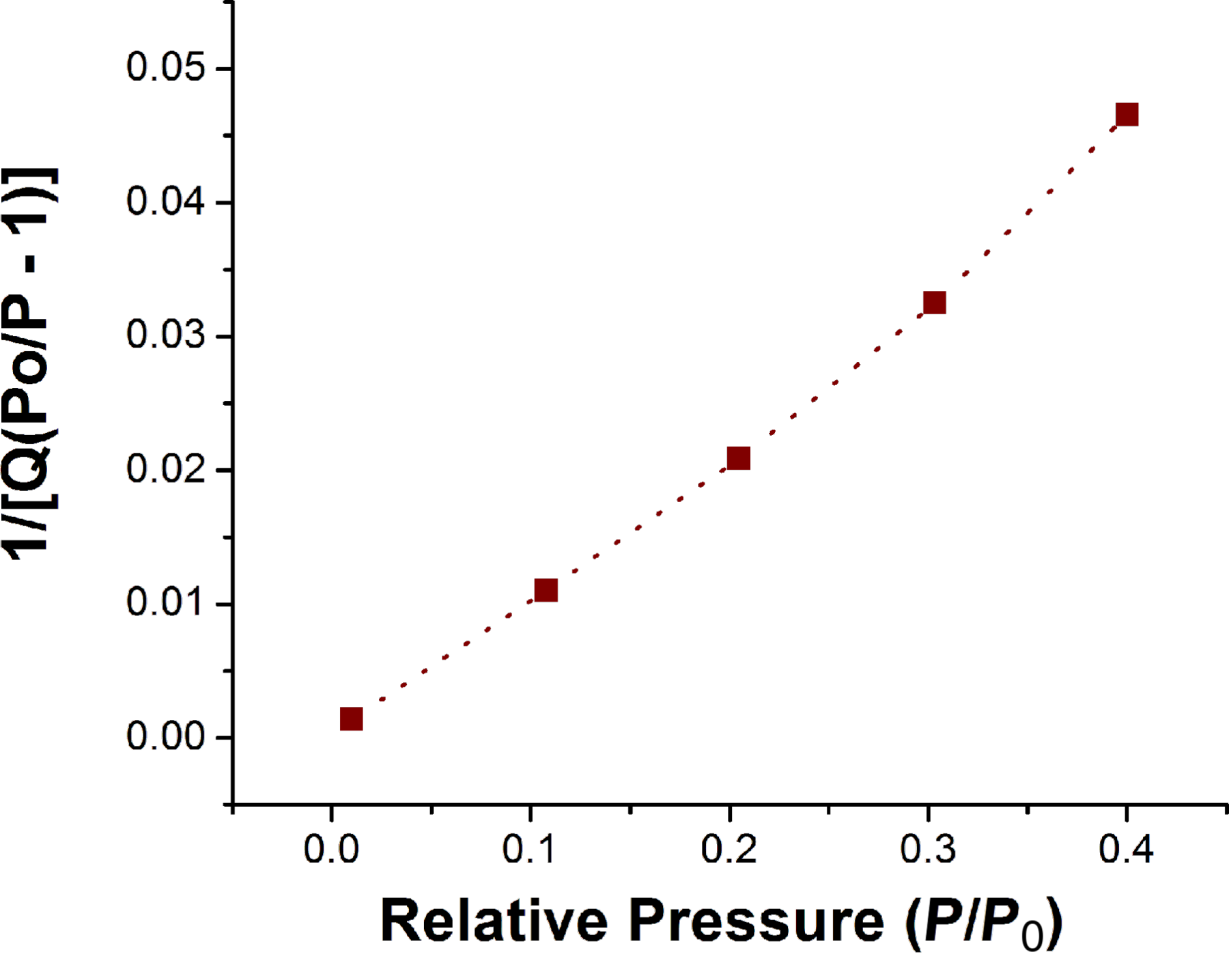
BET surface area plot of the calcined Ta_2_O_5_ nanoparticles.

**Figure 10:**
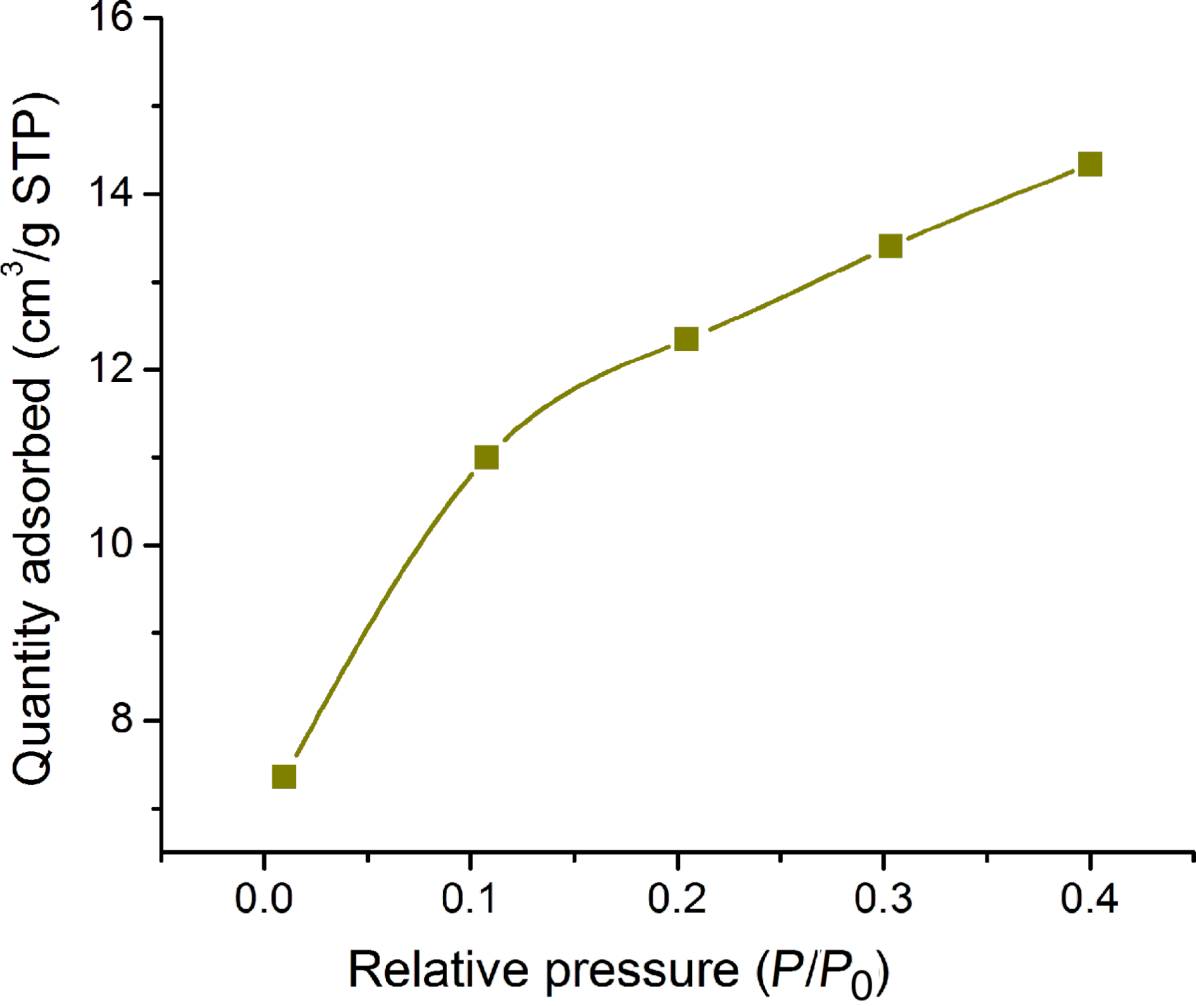
Degradation of rhodamine B by UV irradiation at 0.8 mg/mL catalyst loading.

The BET surface area of the calcined Ta_2_O_5_ nanoparticles was found to be 38.35 m^2^/g. The total volume of pores with diameter less than 32 Å at *P*/*P*_0_ = 0.400214547 was estimated to be 0.022 cm³/g (Figure 9 and Figure 10).

### Photocatalytic experiments

**Figure 11:**
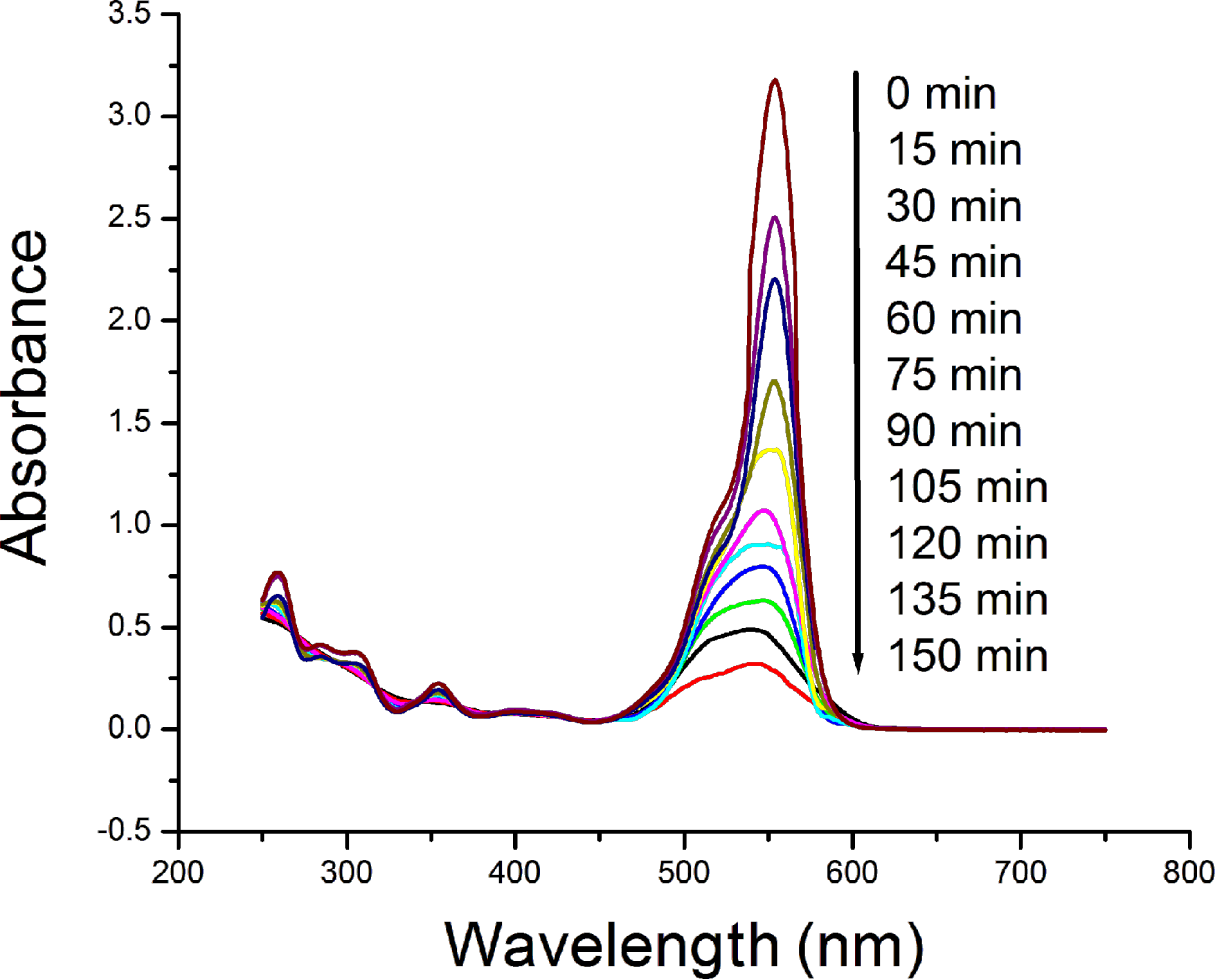
Effect of the concentration of Ta_2_O_5_ nanoparticles on the rate of degradation of rhodamine B.

The photocatalytic activity was evaluated by the degradation of rhodamine B (RhB) under UV radiation for different amounts of Ta_2_O_5_. In each experiment Ta_2_O_5_ was added to 50 mL water and sonicated, followed by addition of RhB and exposure to UV light (λ = 365 nm) irradiation at room temperature. To attain an adsorption–desorption equilibrium, the dispersion was stirred in the dark for 45 min. Just before the irradiation an aliquot of 3.0 mL was taken and centrifuged. The supernatant was taken for recording the absorption spectrum at the initial concentration. After every 15 min, an aliquot of 3.0 mL was taken and the concentration of RhB was measured through the intensity of absorption (Figure 11).

#### Effect of catalyst concentration on the rate of degradation of rhodamine B

**Figure 12:**
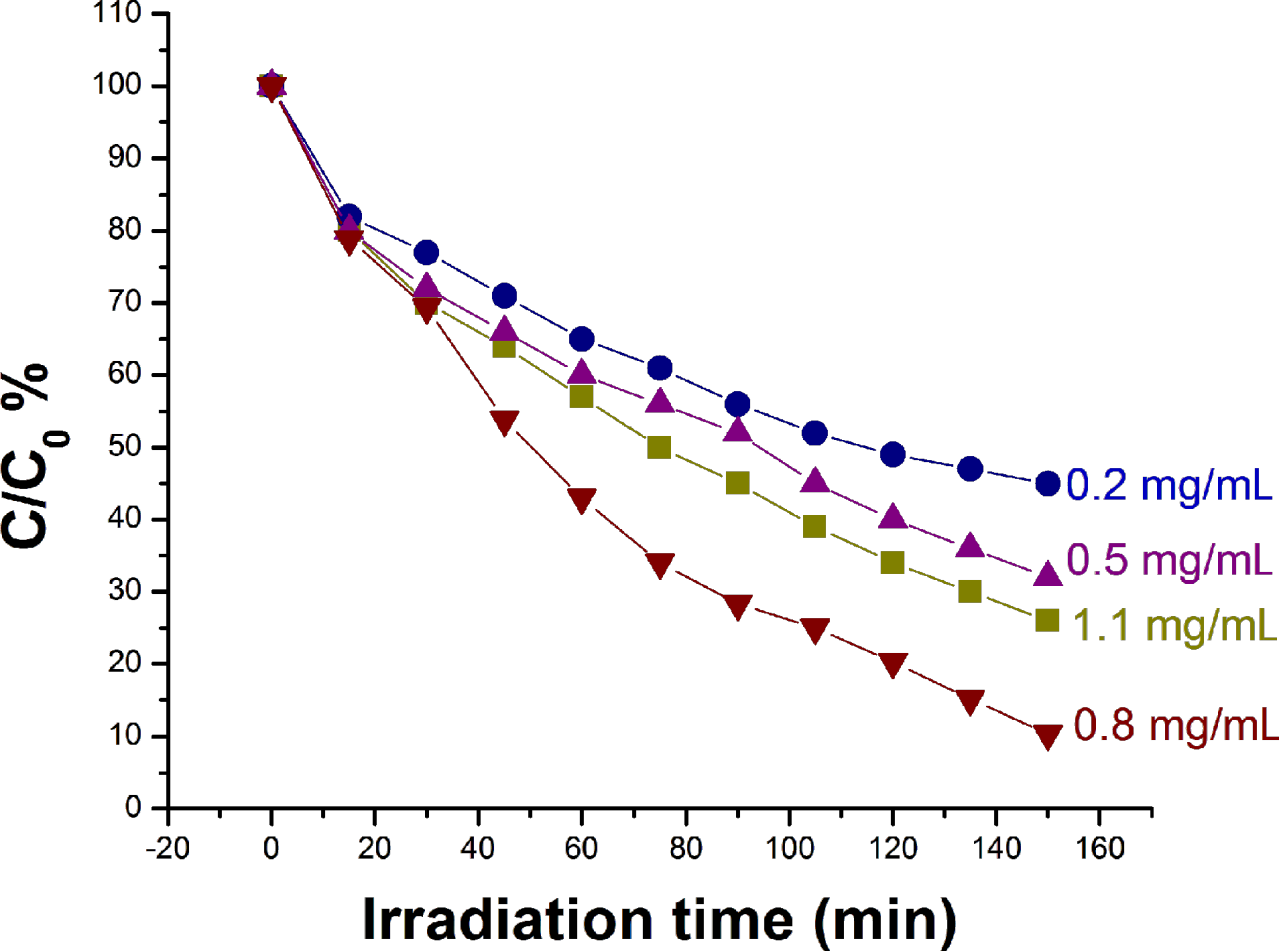
Effect of dye concentration on photocatalytic degradation.

Figure 12 shows the degradation of dye for different of the catalyst loadings. It is clear that the optimum (89%) degradation of the dye was achieved (after 150 minutes) when 0.8 mg/mL of the photocatalyst was used in the experiment. However, when the used amounts of photocatalyst were 0.2 mg/mL, 0.5 mg/mL and 1.1 mg/mL, the degradation of dye occurred up to 55%, 68% and 74%, respectively. The amount of the photocatalyst was changed in each experiment while keeping the other factors invariable to study the optimum degradation of dye with respect to the amount of Ta_2_O_5_. By increasing the amount of Ta_2_O_5_ from 0.2 mg/mL to 0.8 mg/mL, the photocatalytic degradation rate was enhanced due to increase in the active sites accessible for the reaction on the surface of the catalyst. However, when the amount of catalyst was increased further, the rate of dye degradation was found to be lower. This may be due to the scattering of light from surface of the catalyst leading to the reduction in light penetration through the solution, which in turn reduces the rate of formation of radicals. Also, it may be assumed that the activated molecules get deactivated due to the collisions with the ground state molecules and thus reduce the degradation [31].

#### Effect of dye concentration on the rate of degradation of rhodamine B

**Figure 13:**
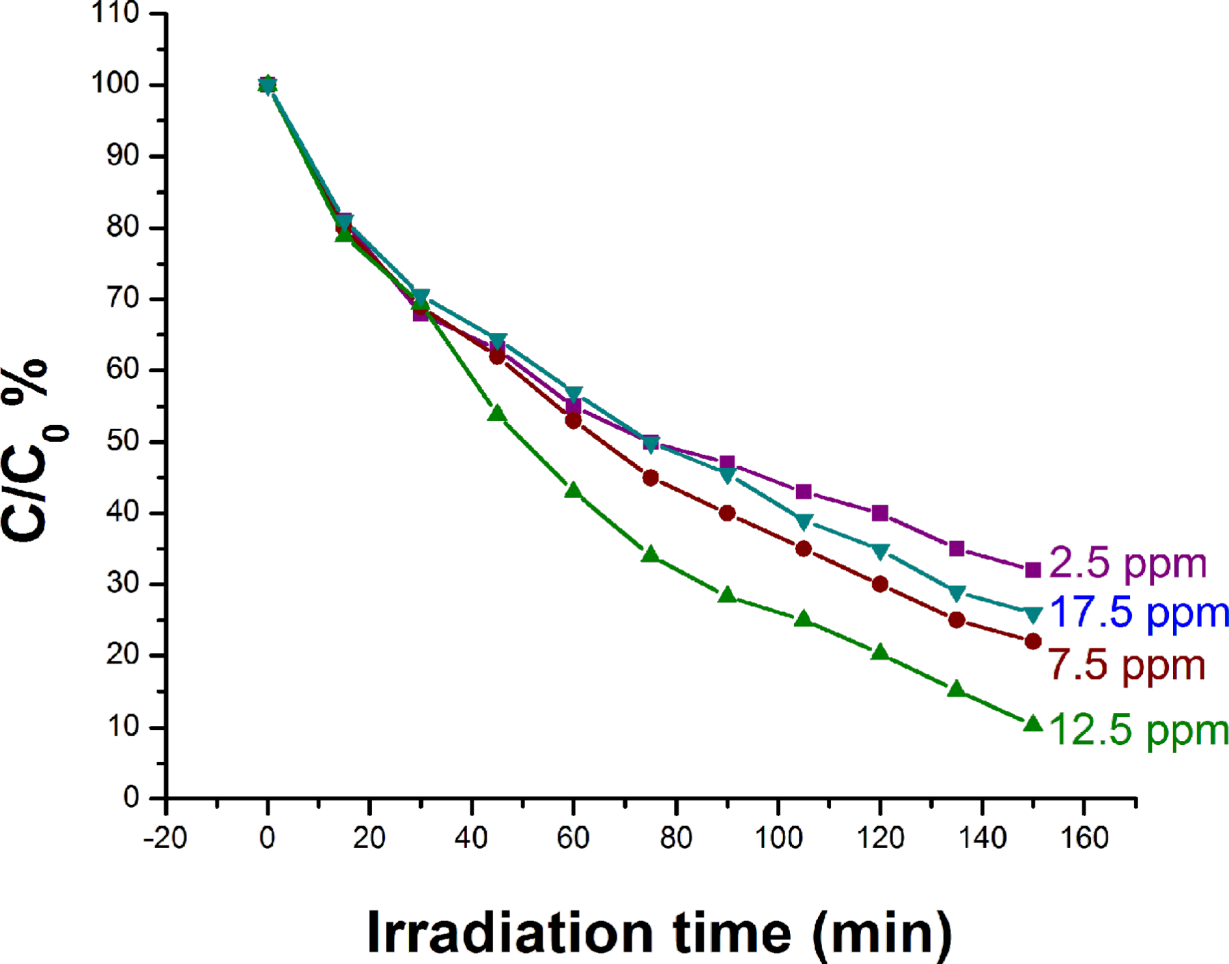
Effect of dye concentration on photocatalytic degradation.

To study the effect of initial dye concentration on the photocatalytic degradation different amounts of rhodamine B were taken while keeping other factors constant. It was observed that on increasing the amount of dye from 2.5 ppm to 12.5 ppm the rate of degradation of the dye increases but a further increase of the dye concentration decreases the degradation rate (Figure 13). With the increase in dye concentration the number of dye molecules available for excitation and intersystem crossing increases, which ultimately increases the rate of degradation. However, when the concentration of the dye was increased beyond a certain limit the excess of the dye serves as a filter for the light and also it does not allow the photons to reach the catalyst surface due to the reduced path length and, hence, retards the rate of degradation.

#### Effect of the pH value on the rate of degradation of rhodamine B

**Figure 14:**
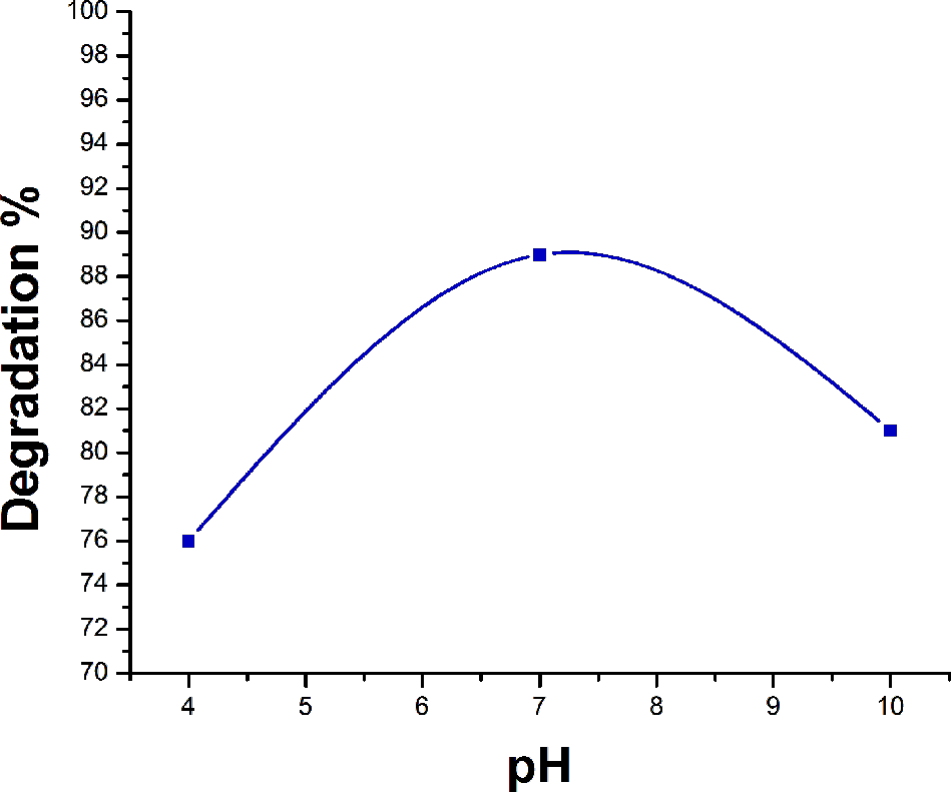
Effect of the pH value on the rate of degradation of rhodamine B.

Figure 14 shows the effect of the pH value on the rate of degradation of rhodamine B. The pH value was adjusted by using dilute solutions of HNO_3_ and NaOH. Degradation of the dye was studied at pH 4, 7 and 10. It was observed that the rate of degradation of rhodamine B increases with the increase in pH from 4 to 7. It appears that, when more hydroxy ions (OH^−^) are available, they combine with the holes (h^+^) of the semiconductor resulting in the production of hydroxyl radicals. These radicals are responsible for the degradation of dye by oxidative process. However, a further increase of the pH value provides excess OH^−^ ions that get absorbed on the catalyst surface and obstruct the approach of the dye molecule to the catalyst surface and slow down the rate of degradation of rhodamine B (Figure 14). Moreover, the metal oxide particles agglomerate at acidic pH and hence the surface available for dye absorbance as well as the photon absorption is reduced.

#### Effect of calcination temperature

**Figure 15:**
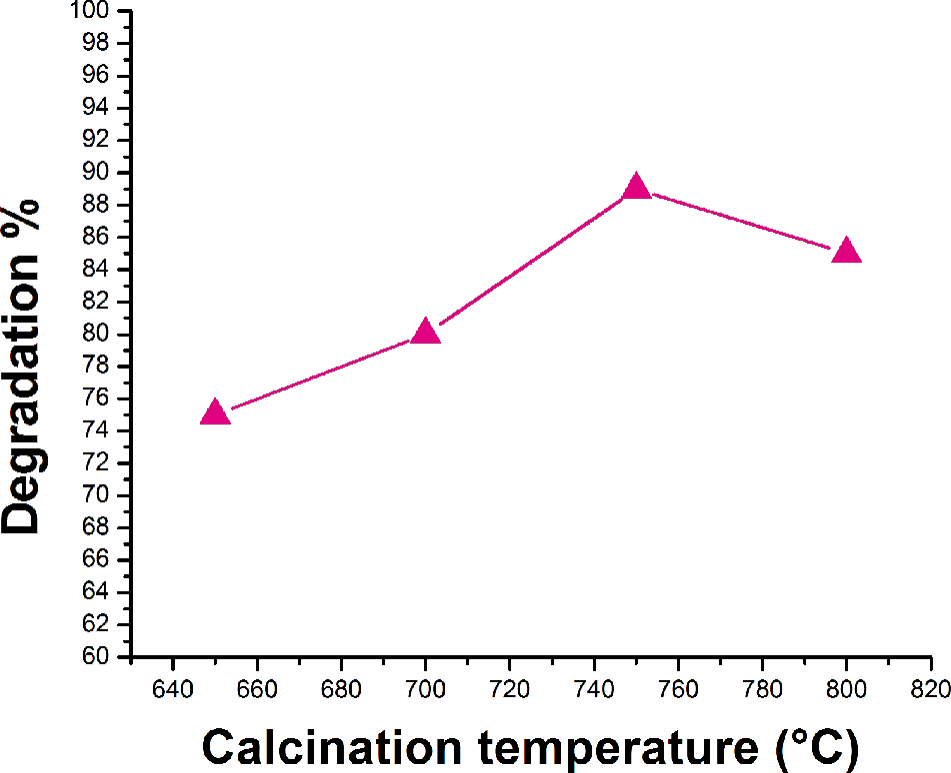
Effect of the calcination temperature on the rate of degradation of rhodamine B.

As-synthesized Ta_2_O_5_ powder was calcined in at 650 °C, 700 °C, 750 °C, and 800 °C. It was observed that Ta_2_O_5_ particles calcined at 750 °C possess best degradation efficiency (Figure 15). This may be due to increase in crystallinity and surface area with more active sites for photodegradation process when temperature is increased from 650 °C to 750 °C. A further increase in calcination temperature leads to bigger particles due to agglomeration at higher temperature, which ultimately reduces the rate of degradation of rhodamine B [32]. At higher temperature the catalyst is deactivated due to sintering processes that result in a low surface area and smaller number of active sites [33].

## Conclusion

In summary, herein we report the formation of the tantalum oxo-ethoxide compound Ta_8_(μ_3_-O)_2_(μ-O)_8_(μ-OEt)_6_(OEt)_14_ (**1**) through a sol–gel route in which hydrous ammonia gas was passed into a solution of tantalum ethoxide in toluene. Ta_2_O_5_ powder was obtained when a complete hydrolysis of **1** was allowed. After calcination the Ta_2_O_5_ nanoparticles were employed for the degradation of the common organic dye rhodamine B. The rate of degradation of the dye highly depends on the various parameters such as amount of the catalyst, dye concentration, pH and calcination temperature.

## Experimental

All the reactions before the catalyst preparation were carried out under anhydrous conditions by using Schlenk/vacuum line techniques. Tantalum ethoxide was purchased from Sigma-Aldrich. Ethanol was dried by standard procedure prior to use. Ammonia gas was first dried by passing through the columns of silica gel, fused calcium chloride and aluminum isopropoxide, then it was passed into the distilled water through a tube with a diameter of 7 mm. ^1^H NMR spectra were recorded in C_6_D_6_ on a Bruker Biospin ARX spectrometer with TMS as internal reference. TGA/DTA/DSC was recorded by using a Diamond TG/DTAN instrument. X-ray diffraction patterns were recorded on SEIFERT XRD 3003 PTS Diffractometer System, using Cu Kα radiation. SEM images was obtained on an EVO MA 15 Zeiss at 15 kV. DLS measurements were carried out on a Nanotrac particle analyser. The surface area was calculated by applying the Brunauer–Emmett–Teller (BET) method to N_2_ adsorption measurements on a Micromeritics ASAP 2020 instrument. TEM pictures were taken on a transmission electron microscope JEOL JEM-1011.

Preparation of **1**: Tantalum ethoxide (100 mg) was dissolved in dry toluene (20 mL). Ammonia gas (passed through 20 mL distilled water at a rate of 30 bubbles/minute) was bubbled into the solution at ambient temperature. After 1 h, a white solid precipitated. The solid was separated, re-dissolved in toluene and kept at −30 °C for crystallization to yield compound **1** in 45% yield (35 mg). ^1^H NMR (25 °C) δ 1.33 (t, CH_3_), 1.41 (t, CH_3_), 1.49 (t, CH_3_), 1.58 (t, CH_3_), 1.62 (t, CH_3_), 4.45 (q, CH_2_), 4.59 (q, CH_2_), 4.70 (q, CH_2_), 4.90–5.00, 5.19 (q, CH_2_), 5.23 (q, CH_2_).

Preparation of Ta_2_O_5_ nanoparticles: Compound **1** was dissolved in toluene and ammonia gas was blown into it for 6 h to get a homogeneous gel. After treating the gel at 80 °C for 3h, a white solid powder was separated out, which was insoluble in any organic solvent. The powder was washed with water and ethanol and dried at 100 °C for 2 h. It was calcined at 750 °C for 4 h to develop crystallinity.

Photocatalytic activity measurements: Typical amounts (0.2 mg/mL, 0.5 mg/mL, 0.8 mg/mL and 1.1 mg/mL) of Ta_2_O_5_ nanoparticles as photocatalysts were taken in 50 mL of distilled water and sonicated for 5 min. Then 12.5 ppm of rhodamine B was added to it. To attain an adsorption–desorption equilibrium between the dye molecules and the catalyst surface, the solution was stirred for about 45 min in dark prior to irradiation. Before exposing to the UV radiation (λ = 365 nm), a 3 mL aliquot was taken, centrifuged and recorded as the zero time concentration of the dye. This process was repeated after every 15 min of exposure to UV light for recording the absorption of the remaining dye.

## Supporting Information

File 1 CIF data of compound **1**.
